# Lung abscess as a complication of Lemierre Syndrome in adolescents: a single center case reports and review of the literature

**DOI:** 10.1186/s13052-023-01499-4

**Published:** 2023-08-10

**Authors:** Laura Venditto, Giuliana Ferrante, Anna Caccin, Giulia Franchini, Marco Zaffanello, Laura Tenero, Michele Piazza, Stefano Di Gioia, Giorgio Piacentini, Angelo Pietrobelli

**Affiliations:** 1 Department of Surgical Sciences, Dentistry, Gynecology and Pediatrics, University of Verona, Verona, Italy; 2 Pediatric Division, University Hospital of Verona, Verona, Italy; 3 Department of Otolaryngology-Head and Neck Surgery, University Hospital of Verona, Verona, Italy

**Keywords:** EVALD, Fusobacterium necrophorum, Lemierre syndrome, Lung abscess, Case report, Adolescents

## Abstract

**Background:**

*Fusobacterium necrophorum* is an anaerobic, gram-negative, non-motile, filamentous, non-spore forming bacillus found in the oral cavity, gastrointestinal tract, and female genital tract, responsible of a rare disease named Lemierre Syndrome, characterized by septic thrombophlebitis of the internal jugular vein, which mainly affects previously healthy adolescents and young adults; some risk factors are reported, as smoking or primary viral or bacterial infection leading to the disruption of mucosa. The syndrome originates commonly from an upper respiratory infection such as pharyngotonsillitis, acute otitis media, cervical lymphadenitis, sinusitis, or odontogenic abscess, and may result in multiorgan metastasis, more frequently leading to pulmonary complications, especially lung abscesses.

**Case presentation:**

We describe two cases of adolescents with atypical Lemierre Syndrome evaluated in a tertiary care center, one with a confirmed infection by *Fusobacterium necrophorum* and one with a presumptive diagnosis based on clinical features, who developed lung abscesses needing a prolonged antibiotic course and hospitalization. Of interest, both were user of electronic cigarette, configuring a possible new risk factor. The proper diagnosis of Lemierre Syndrome is often difficult to establish, so a high degree of suspicion is needed, especially in the case of lung abscesses in otherwise healthy adolescents.

**Conclusion:**

The current study will contribute to providing insight into Lemierre Syndrome clinical presentation and management in adolescents, promoting awareness for a rare but potentially fatal disease. Moreover, it suggests a possible relationship between Lemierre syndrome and the use of electronic cigarette, that should be investigated by future studies.

**Supplementary Information:**

The online version contains supplementary material available at 10.1186/s13052-023-01499-4.

## Background

Lemierre Syndrome (LS) is a rare disease characterized by septic thrombophlebitis of the internal jugular vein (IJV), primarily due to *Fusobacterium necrophorum* (FN), which affects previously healthy adolescents and adults. The syndrome originates from an upper respiratory infection that spreads to the IJV, causing septic thrombophlebitis, which results in multiorgan metastasis, and more frequently pulmonary complications.

The average length of stay in the hospital is approximately three weeks. Septic emboli and end-organ effects can result in long-term morbidity [1]. Even with appropriate antibiotics and therapy, mortality has been reported to be between 5% and 18% [1].

Therefore, a timely diagnosis, close clinical/imaging monitoring and a multidisciplinary management appear necessary [2].

We report two cases of atypical LS in adolescents who developed lung abscesses needing a prolonged hospitalization. Of interest, both were user of electronic-cigarette (e-cigarette), configuring a possible emerging risk factor for LS.

A narrative literature review was conducted. We searched original papers in English in the PubMed database using the following keywords, separately and in combination: Lemierre syndrome; lung abscess; Fusobacterium necrophorum; Fusobacterium; septic thrombophlebitis. Age restrictions were set to child (birth − 18 years). No limitations were set for the date and study country. We also consulted the reference lists of the retrieved articles.

### Case presentation

A 14-year-old girl was admitted to the emergency room for presenting fever for five days, headache, and sore throat. Active smoking was reported. At admission, the girl appeared asthenic, pale, tachycardic, and febrile at the examination. Hyperemic pharynx and bilateral cervical adenopathy were observed. Ear-Nose-Throat (ENT) evaluation showed hyperemia of the rhino-pharynx-laryngeal cavity. Laboratory tests showed normal leukocyte count (leukocytes 7170/mm^3^), unchanged formula (neutrophils 5430/mm^3^), and increased levels of C-reactive protein (CRP, 134 mg/L). Chest X-ray and an ultrasound of the abdomen were both normal. Rapid Group A *Streptococcus pyogenes* test was negative.

**Fig. 1:**
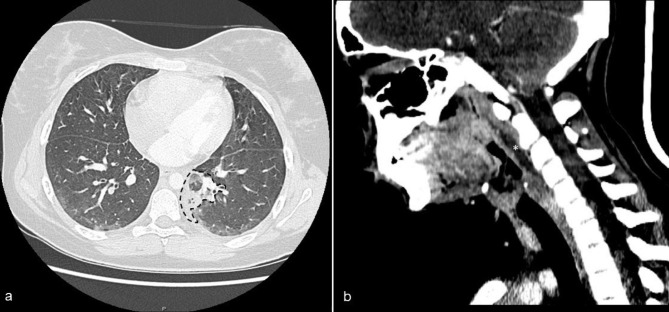
Axial, contrast-enhanced CT scan detecting a lung abscess of 3 cm in the left inferior pulmonary lobe (dashed line) (**a**). Sagittal, Contrast-enhanced CT scan showing a retropharyngeal abscess (*) (**b**)

Antimicrobial therapy with ceftriaxone was started, and the girl was admitted to the hospital ward, where she had a rapid decline in her condition, with hypotension and respiratory distress. Blood laboratory exams showed increased D-Dimer concentration (16,814 ug/L). For this reason, low-molecular-weight heparin (LMWH) was started, and a thoracic CT angiography was performed that excluded thromboembolisms, detecting instead a lung abscess of 3 cm in the inferior left pulmonary lobe (Fig. 1a).

At day + 2, no improvement was observed; meanwhile, the blood culture reported a gram-negative infection, so ceftriaxone was switched to piperacillin/tazobactam.

At day + 3, the blood culture showed the growth of a FN. In the suspicion of LS, an ultrasound of neck veins did not show thrombosis of the IJV (IJVT). A neck CT angiography showed a peritonsillar left abscess and a retropharyngeal abscess with no evidence of IJVT (Fig. 1b); this finding was consistent with diagnosis of atypical LS.

The clinical response was progressive with normalization of blood count, CRP, and D-dimer. Therefore, LMWH was stopped, intravenous antibiotic therapy was switched to oral metronidazole, and the girl was later discharged. The antibiotic regimen was continued for three weeks, after those a chest CT scan was performed, showing a consistent regression of the lung abscess.

**Fig. 2:**
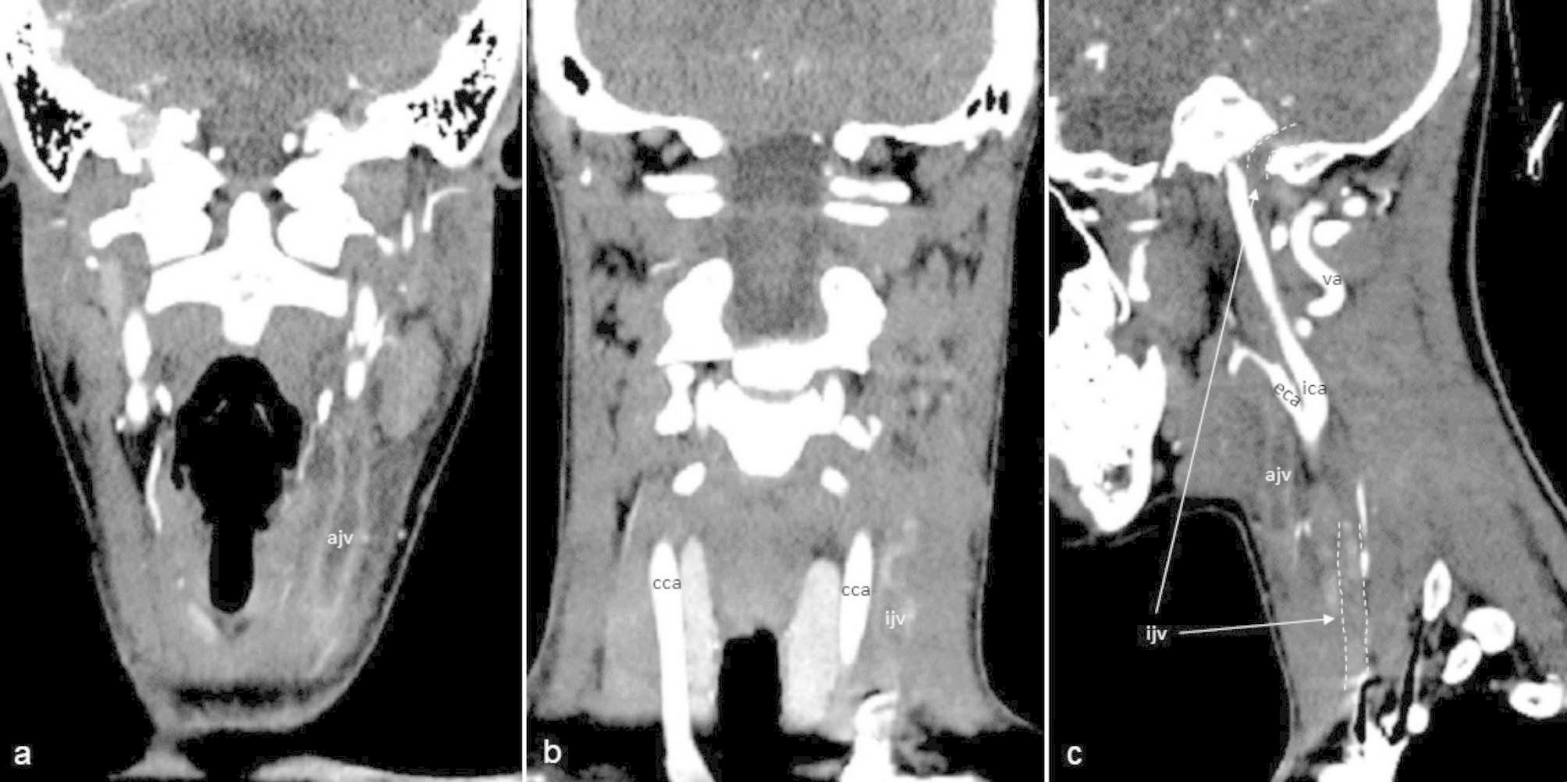
Coronal, post-contrast CT scan showing the left anterior jugular vein and left internal jugular vein thrombosis (**a**-**b**). Sagittal, contrast-enhanced CT scan showing the major blood vessels on the left side of the neck, the anterior jugular vein, and internal jugular vein thrombosis (**c**). *ajv: anterior jugular vein; cca.: common carotid artery; eca: external carotid artery; ica: internal carotid artery; ijv: internal jugular vein; va: vertebral artery*

**Fig. 3:**
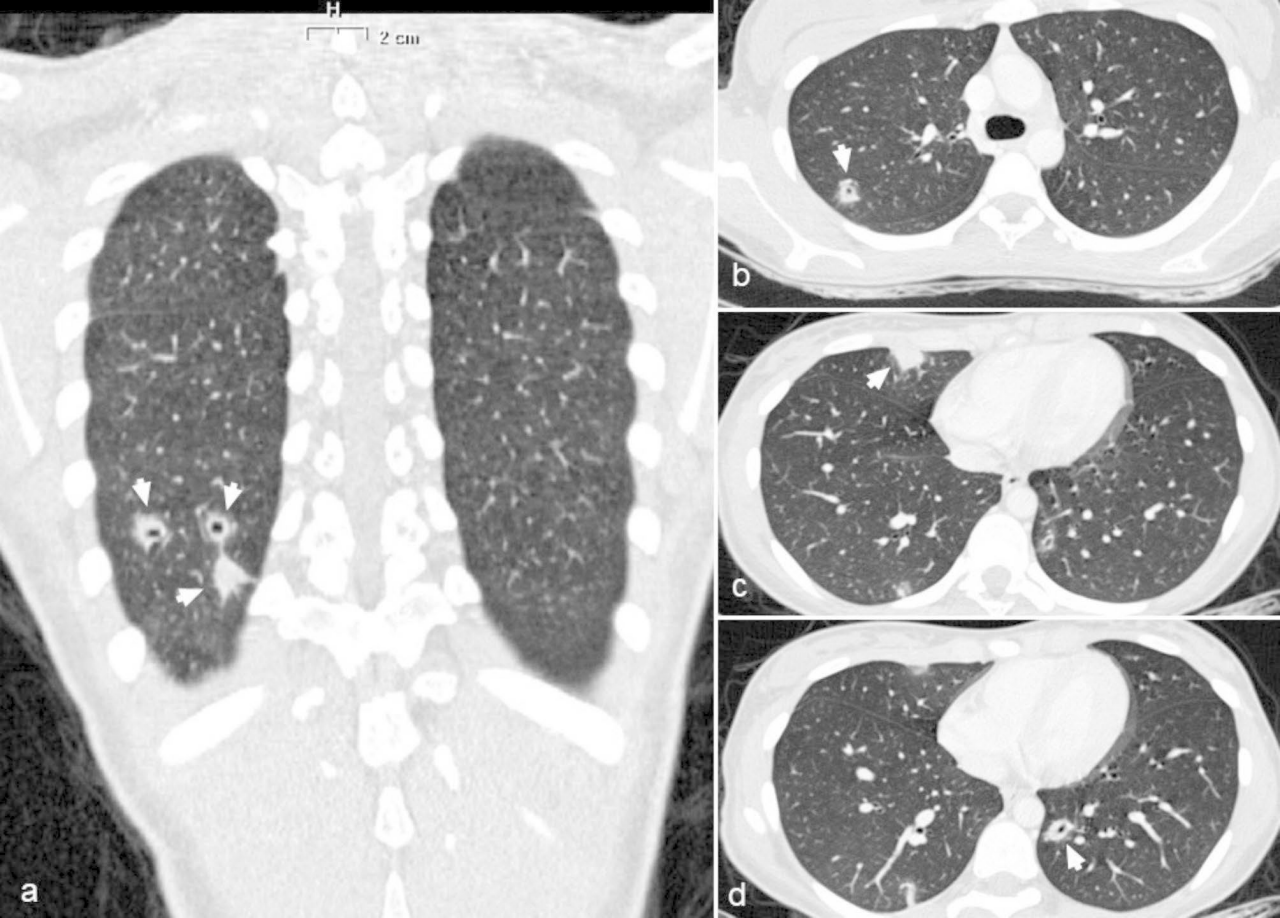
Contrast-enhanced CT scan of the thorax showing multiple lung abscesses (arrowhead) located in the right lower lobe (**a**); right upper lobe (**b**); middle lobe (**c**); left lower lobe (**d**)

The second case is a 14-year-old girl, an active smoker, who presented sore throat and fever for 9 days, not responsive to clarithromycin and amoxicillin-clavulanic acid, before being admitted to emergency department. She also reported thoracodorsal pain exacerbated by deep breaths. Familiar history was positive for myocardial infarct at young age. A left tonsil enlarged was found at the examination with associated left neck lymphadenopathy. The blood exams showed leukocytosis (13.630/mm^3^) and increased CRP levels (69 mg/L). The electrocardiogram and the chest X-ray resulted in normal. A neck ultrasound confirmed the left lymphadenopathy. Ceftriaxone was started; at day + 1, a left neck mass was noted, and a neck ultrasound showed multiple thromboses of the superficial veins of the neck. A neck CT was then performed, showing left IJVT that involved the intracranial portion (Fig. 2), enabling the diagnosis of atypical LS. LMWH was started, and metronidazole was added to the ongoing antimicrobial treatment. A thorax CT showed multiple septic pulmonary emboli (Fig. 3). Gradual clinical improvement was observed, allowing an antibiotic shift to amoxicillin-clavulanic acid at day + 10. The blood cultures resulted negative, probably due to the antibiotics administered before the admission. The patient was then discharged. A neck and thorax CT was repeated after 3 weeks, detecting a complete resolution of pulmonary infiltrates and of the IJVT. Antibiotic therapy and LMWH were continued for 4 weeks and three months respectively. The patient was compliant with the therapy, which was well-tolerated and monitored with Anti factor Xa assay. No adverse effects were observed during the follow-up.

### Discussion and conclusions

We reported two cases of atypical LS in female adolescents, who both developed lung abscesses, whose clinical features are summarized in Table 1.

**Table 1:** Clinical characteristics of the patients

	Case 1	Case 2
**Sex**	F	F
**Age**	14	14
**IJVT**	no	yes
**Blood culture**	*Fusobacterium necrophorum*	negative
**Primary infection**	peritonsillar left abscess and retropharyngeal abscess	pharyngotonsillitis
**Septic emboli**	yes	Yes
**Locations**	lung	Lung
**Symptoms**	5-day fever, sore throat, headache, cervical pain	9-day fever, sore throat, thoracodorsal pain
**Intravenous antibiotic**	piperacillin/tazobactam	ceftriaxone and metronidazole
**Oral antibiotic**	metronidazole	amoxicillin-clavulanic acid
**Anticoagulant**	prophylactic LMWH	therapeutic LMWH
**Risk factors**	active smoke	active smoke
**WBC on hospital admission [mm³]**	7170 (NR 4500–13,000)	13,630 (NR 4500–13,000)
**Neutrophil granulocytes on hospital admission [mm³]**	4530 (NR 1800–80,000)	NA (NR 1800–80,000)
**C- reactive protein on hospital admission [mg/L]**	134 (NR < 5)	69 (NR < 5)
**D-dimer on hospital admission [µg/L]**	16,814 (NR < 500)	393 (NR < 500)
**Chest X-ray**	no pathological finding.	no pathological finding.
**Abdominal Ultrasonography**	no pathological finding.	no pathological finding.
**Thorax CT**	single septic pulmonary embolus	multiple septic pulmonary emboli
**Thorax CT at follow-up**	resolution	resolution

IJVT: Internal jugular vein thrombosis; LMWH: Low-Molecular-Wight Heparin; WBC: White blood cells; NR: normal reference; CT: computer tomography

LS is a rare disease, whose incidence was 14.4 cases/million/year in adolescents and young adults [3]. The patients described were adolescents, with a median age reported in the literature between 19 [4] and 21 years [2]. Moreover, although LS occurs predominantly in healthy male adolescents [5–7], with a man-to-woman ratio of 2:1 [8], our cases were both female.

In details, in the last 20 years, 16 cases of LS in adolescents have been reported in literature (Table 2), with a median age of 15.5 years. Males were more frequently involved (62.5%). No particular risk factors were reported, but interestingly one had a history positive for vaping, and 18.75% had thrombophilia; ENT infections such as pharyngitis, peritonsillar abscess, and sinusitis were the most common primary infection (81.25%). A thrombus in the IJV was identified in 87.5% of cases, whereas Fusobacterium species were found in 81.25% of cases. Lung involvement with septic emboli, necrotizing pneumonia, pleural effusion was present in most of the cases (93.75%).

**Table 2:** Lung involvement in adolescents with Lemierre syndrome reported in the literature in the last 20 years

Authors and year	Gender/Age (years)	Risk Factors	Primary site of infection	Site of the thrombus	Microbiology	Lung involvement	Antibiotic treatment	Outcome
El Chebib et al (9) 2020	F, 15	Vaping	None	Not identified	FN	Necrotizing pneumonia with cavity and parapneumonic effusion	Piperacillin plus tazobactam	Exertional dyspnoea at one month
Repper et al. (10) 2020	F,15	Family history of thrombosis	Neck stiffness and trismus	Right and left IJV	FN	Bilateral pulmonary infiltrates and pleural effusion	Ceftriaxone plus clindamycin, then shifted to meropenem; then amoxiclavulanate	Good
Sheth et al (11) 2018	M,17	None	Left frontal sinusitis, complicated by subdural empyema, subperiosteal abscesses and osteomyelitis of the lateral orbital rim	Right IJV	Fusobacterium nucleatum	Multiple pulmonary nodules with cavitation	Vancomycin plus metronidazole, then shifted to ceftriaxone plus metronidazole	Good
Unic et al (12) 2018	F,17	Ulcerative colitis	Acute pharyngitis	Right subclavian vein, right jugular veins, truncus brachiocephalicus, partial thrombosis of the right sigmoid and transverse sinus	Not identified	Bilateral pleural effusion	Ceftriaxone plus clindamycin	Good
Monroe et al (13) 2018	M,17	None	Acute pharyngitis	Left jugular vein and dural venous sinus, complicated by left cervical carotid stenosis with a mycotic pseudoaneurysm	FN	Septic emboli	Intravenous antibiotics, not specified	AAt 4 months thrombosis of the pseudoaneurysm
Kumral et al (14) 2017	F,14	None	Acute pharyngitis	Left IJV	FN	Multiple bilateral nodules	Ceftriaxone, then shifted to piperacillin plus tazobactam	Good
Bonhoeffer et al (15) 2010	F,15	None	Pharyngotonsillitis and peritonsillar abscess	Left IJV	FN	Multiple nodular infiltrates in the lower lobes	Amikacin, cefotaxime, and metronidazole; then amoxiclavulanate	Not reported
Ridgway et al (5) 2010	M,16	None	Right peritonsillar and parapharyngeal abscess, right mastoiditis	Right IJV	FN	Septic emboli	Clindamycin, ceftriaxone, and azithromycin; then ceftriaxone, metronidazole, and penicillin G	Relapse 14 months later
Ridgway et a (5) 2010	M,17	None	Acute pharyngitis	Right IJV	FN	Bilateral cavitary nodules, pulmonary effusion, and atelectasis	Cefotaxime plus vancomycin	Not reported
Horwitz et al. (16) 2013	M,14	None	Pansinusitis	Venous sinus	Methicillin-susceptible Staphylococcus aureus	Necrotizing pneumonia	Clyndamicine plus linezolid	Good
Hawes et al (17) 2013	M,17	None	Pharyngotonsillitis	Not investigated	Fusobacterium spp.	Left-sided pleural effusion	Cefuroxime plus erytromicin, and metronidazole, shifted to amoxiclavulanate	Good
Waterman et al(18) 2007	F,15	None	Bronchitis	Common jugular vein and right jugular vein	Not identified	Cavitary lesions and nodular opacities in the right upper lobe	Clindamycin plus ceftriaxone	Good
De Lima et al (19) 2003	M,15	None	Pharyngotonsillitis	Both IJV	Fusobacterium	Multiple bilateral nodules, with cavitation, and pleural effusion	Intravenous antibiotics, not specified	Not reported
Busko et al (20) 2004	M,14	Immobilization for 6 weeks for trauma	Acute pharyngitis	Right IJV	FN	Multiple bilateral emboli	Vancomycin, gentamycin and clindamycin	Good
Hoehn et al (21) 2002	M,17	None	Acute gastroenteritis	Left IJV	FN	Multiple nodules, and pleural effusion	Ampicillin-sulbactam plus metronidazole, then amoxiclavulanate	Good
Klinge et al (22) 2002	M,13	APC resistance with Factor V Leiden mutation	Retropharyngeal abscess and left otitis media, complicated by osteomyelitis of the left part of the atlas and clivus	Left sigmoid sinus and left IJV	Fusobacteria	Not reported	Meropenem, vancomycin and gentamycin, then oral penicilline	Good

The peak of incidence usually occurs in winter [7], although some studies have not shown a seasonal prevalence [4, 7]. Interestingly, we have observed two cases over a three-month period, between April and July 2022.

In the last 30 years, there has been an increase in hospitalizations for Fusobacterium spp (FS) infections in children, possibly due to the decrease in tonsillectomies, the use of corticosteroids for infectious mononucleosis, the decreased use of antibiotics in ENT infections, and the improvement of microbiological techniques [23]. Another potential factor is the use of antibiotics to which FN is resistant. Indeed, resistance to erythromycin is common in FN, and some hospitalized patients had previously been treated with macrolides or erythromycin [7, 24, 25].

Nowadays, mortality is estimated at 4–12%, increasing in case of therapeutic delay [26], due to brain involvement (meningitis, abscess, cerebral edema), mediastinitis, retrosternal abscess, disseminated intravascular coagulation, and septic shock [4].

In most cases, the primary infection involves tonsils and peritonsillar tissue, but odontogenic infection, sinusitis, otitis media, mastoiditis, and parotitis have been reported [5, 23], with an age distribution: in children, it usually originates from neck lymphadenitis or acute otitis media, in adolescents from pharyngotonsillitis, whereas in adults from sinusitis and odontogenic infections [23]. The primary infections are usually caused by members of the normal oropharyngeal flora [27]; the most common is FN [4, 5, 28], which is an anaerobic, gram-negative, non-motile, filamentous, non-spore-forming bacillus found in the oral cavity, gastrointestinal tract, and female genital tract [8, 23, 29]. FN has multiple virulence factors including the cell wall lipopolysaccharide endotoxin, lipases, leucocidins, DNAases, hemolysins, hemagglutinins, neutrophil cytotoxic factors, and the ability to aggregate platelets [5, 23]. Other pathogens are *Fusobacterium nucleatum* [5], Bacteroides [30], Enterobacteriaceae [5, 31], *Eikenella corrodens* [5, 32], *Streptococcus pyogenes* [33]^,^*Streptococcus anginosus* [34], rarely also *Staphylococcus aureus*, including methicillin-resistant S. aureus [35, 36]. In some cases it has not been possible to identify the pathogen [5], as in our second case, probably due to antibiotics administered before admission.

The pathogenesis of LS is multifactorial, including host predisposing factors, viral and bacterial co-infections, and bacterium virulence factors [5, 6, 23, 29, 37]. The local invasion by FN may occur because of the oropharyngeal mucosa disruption that leads to hypoxia, creating the environment necessary for bacterial proliferation [23, 29, 37]. The mucosal alteration can be secondary to accidental or surgical local trauma, primary viral or bacterial infections, and chronic nicotine irritation [8, 23]. Notably, our patients had an early habit of smoking and vaping, which could be acknowledged as an emerging risk factor for LS in adolescents [9]. Some authors have reported an association with Epstein-Barr virus pharyngitis, but the mechanism remains uncertain [5, 6, 29, 37–39].

Septic thrombophlebitis could occur for a hematogenous spread via the tonsillar vein, peritonsillar tissue invasion, and secondary spread to the adjacent lateral pharyngeal space via lymphatics; alternatively, tonsil abscesses spread into the connective tissue of the pharynx causing purulent phlebitis [5, 8]. Possible metastatic foci may occur due to the direct invasion of the pathogens into the blood system or via septic emboli [5, 23].

Pediatricians should be aware of the first clinical manifestations of LS that are frequently preceded by signs and symptoms of the initial infection as a pharyngotonsillitis with hyperemic, exudative, ulcerated, but even normal tonsils [5, 8, 40], or resolved by the time thrombotic and septic complications occur, which generally happens after 1–3 weeks [8, 27], as in our cases. Symptoms are typically fever, rigor, sore throat, dysphagia, and unilateral neck pain [5, 8]. Signs of IJVT include unilateral neck swelling, tenderness at the ipsilateral angle of the mandible or induration over the neck or along the sternocleidomastoid muscle, and limitation of the head’s movements, but, in view of the fact that local signs could be absent [8, 28], ultrasonography is useful to detect the thrombus that appears hyperechoic with decreased or absent blood flow; the doppler ultrasound may demonstrate loss of respiratory phasicity and cardiac pulsatility of the IJV, which indicates a more proximal thrombus in a vein that is not accessible with ultrasound [41]. However, even the ultrasound could miss the thrombus due to coexistent edema or adenopathy; in such cases, contrast-enhanced CT (CECT), or Magnetic Resonance Imaging (MRI), may be required to confirm the presence of a thrombus and characterize the extent of disease [42].

Manifestations of metastatic foci of infection may be present at the time of initial presentation or develop later. Lungs are commonly involved, so clinicians should include LS in the differential diagnosis in patients with signs related to septic emboli, as dyspnea, cough, respiratory distress, chest pain [43]; children usually present a low-grade fever, and cough, while chest pain, and hemoptysis are less common. The pulmonary manifestations vary in severity, ranging from nodules with or without a necrotic cavity, pleural effusion, to necrotizing pneumonia, empyema, and abscesses [28], as it can be deduced from Table 2. Other complications could include bronchopleural fistula, pneumothorax, lung consolidation, and mediastinal shift [44]. Septic pulmonary emboli could be seen on chest X-ray as nonspecific peripheral infiltrates, but CECT could better detect them [41] as filling defects in the vessels [45]. Notably, non-CECT septic thrombi appear as an intravascular hypo- and hyper-attenuation, but they could not be seen in some cases as severe anemia [45].

Lung abscesses are usually detected by chest X-ray, which shows a well-demarcated, thick-walled cavity with air-fluid level [44]; a thorax CT is indicated in case of doubt or when no improvement is seen after appropriate treatment.

The second most common sites affected by septic emboli are the large joints such as hips, shoulders, and knees [8, 28] with manifestations that range from arthralgia to septic arthritis, rarely osteomyelitis. Other complications include splenic and liver abscesses, endocarditis, pericarditis, soft tissue abscesses, and septic shock [8, 30, 43]. Central nervous system (CNS) complications are unusual; they include meningitis, subdural empyema, cerebral infarction, and cerebral venous thrombosis as the result of a retrograde extension of thrombosis [8]. In the second case presented, the CT showed intracranial extension of the thrombosis, but the patient did not develop CNS signs.

It’s important to run a full laboratory panel for hypercoagulability that could show thrombophilic abnormalities, in particular elevated factor VIII activity, and the presence of antiphospholipid antibodies [46], which may represent a result of the inflammatory prothrombotic process of LS. In our cases, a study of hypercoagulability was later performed after the stop of LMWH, and resulted normal.

Due to the rarity of the syndrome, the inexperience of clinicians, and the absence of an international consensus [40] the diagnosis of LS appears challenging; however, three criteria could be considered to make diagnosis of LS [8, 29, 40, 47]:

1. Consistent site of primary infection.
2. Isolation on blood cultures of causative pathogen, mainly FN.
3. IJVT with or without metastatic complications.

In patients who partially satisfy these criteria, a presumptive diagnosis of “variant”, “atypical” or “incomplete” LS may be suggested. For instance, in the first case, we could not demonstrate the IJVT, perhaps due to the prompt administration of LWMH before performing neck CT; in the second case, the antibiotic treatment had been started before the hospital admission, probably invalidating the blood cultures.

The management of LS in children and adolescents should be multidisciplinary, involving pediatricians, infectious disease specialists, radiologists, ENT specialists, and pediatric surgeons.

Treatment usually requires appropriate antimicrobial therapy. Nonetheless, no trials have been conducted so far to choose the best antibiotic regimen. FN is susceptible to penicillin, although some strains may be beta-lactamase producers. However, the empiric antibiotic regimen should cover FN and other potential pathogens involved. A systematic review [48] reported that the most common regimen was carbapenem and piperacillin/tazobactam, either as monotherapy or in combination with metronidazole with a duration ranging from 10 days to 8 weeks. These data have been confirmed in a more recent meta-analysis [49], in which an average length of 33.7 days of antimicrobial therapy (range 14–60 days) was reported. In the occurrence of a lung abscess, recommended empiric regimens are piperacillin-tazobactam, meropenem, or second-generation cephalosporin plus metronidazole [50]. Macrolides have shown to be effective against polymicrobial bacteria in lung abscesses, except on FS; likewise, aminoglycosides are not recommended because they poorly pass into the lung abscess [50]. The duration of the antibiotic therapy is controversial, but it seems reasonable to establish a course of at least three weeks [44].

Importantly, LS antimicrobial treatment should be individualized according to antibiotic sensitivity. Generally, clinical improvement can be seen in one week, allowing to switch to the oral route after two weeks of intravenous antibiotics [4]; metronidazole is usually the first choice, thanks to its activity against FN, good penetration in the tissue, and good oral availability [4].

Nonetheless, the response to the antibiotic could be poor and slow due to the difficult penetration of the antibiotic into the fibrin clot, like in abscesses, and necrotizing tissue [51]. In these cases, surgical intervention with debridement of devitalized tissue and incision or drainage of the abscess [23] seems to be reasonable [4]. The ligation and resection of the IJV [23] may be considered in cases of uncontrolled sepsis or septic embolism despite antibiotic treatment [8].

Along with antimicrobial therapy, anticoagulation should be considered to prevent the progression of the thrombus, the septic embolism, and the recurrent venous thromboembolism, but its use remains controversial due to the lack of high-quality evidence [47], especially in pediatric age. The choice to use anticoagulants depends on an individual basis and the preference of the team. The most documented molecule is LMWH, which should be considered in patients with no or slow response to the antimicrobial therapy, given the increased risk of systemic thrombosis and in the absence of any presumed risk of use or contraindications [47]. In a single-institution case series of 9 children with LS [46], all the patients presented a transient state of thrombophilia with antiphospholipid antibodies and elevated factor VII activity, resolved in all cases during the follow-up, suggesting an epiphenomenon of the acute inflammatory prothrombotic state. These children were given anticoagulants (median duration three months); a radiological follow-up showed no thrombus progression and complete resolution in 4 patients, whereas thrombosis persisted in 4 patients (44%) who had manifested a completely vaso-occlusive IJVT. This could lead to the assumption to consider anticoagulation in cases where IJVT is complete at diagnosis. In a 10-year, single-institution retrospective study [42] of septic thrombophlebitis in 28 children, 57% of the patients had complete resolution of thrombus; persistent thrombus was common in septic thrombophlebitis of neck locations, particularly involving the IJV. The resolution of thrombus occurred in 48% and 50% of patients at the end of anticoagulant and antibiotic treatments, respectively. Concerning treatment duration, current guidelines [52] suggest administering (Grade 2 C) anticoagulant therapy in children with secondary venous thromboembolism (VTE) (i.e., VTE that has occurred in association with a clinical risk factor) in whom the risk factor has resolved, as in LS, for three months. Nonetheless, a recent randomized clinical trial [53] has proved that in patients younger than 21 years of age with provoked VTE, anticoagulant therapy for six weeks is not inferior compared with three-month treatment, considering recurrent VTE risk and bleeding risk. According to these findings, it could be considered to shorten the duration of anticoagulation from three months to six weeks, even in pediatric patients with LS.

In conclusion, we highlight the importance of considering LS, especially in adolescents with age-related risk factors, as premature smoking habit and use of electronic cigarettes that could lead to severe pulmonary involvement configuring an electronic cigarette or vape product use associated lung disease (EVALD) [54].

Open issues in the management of LS are the choice of the most appropriate antibiotic and anticoagulant regime and their duration; given the rarity of LS, multicentric randomized clinical trials, and international consensus are needed to better define and standardize the clinical care.

### Electronic supplementary material

Below is the link to the electronic supplementary material.

Supplementary Material 1

## Data Availability

Data sharing is not applicable to this article as no datasets were generated or analysed during the current study.

## Abbreviations

CECT: Contrast-enhanced CT
CNS: Central nervous system
CRP: C-reactive protein
LS: Lemierre Syndrome
IJV: Internal jugular vein
*ENT*: *Ear-nose-throat*
EVALD: E-cigarette or vape product use associated lung disease
FN: *Fusobacterium necrophorum*
FS: Fusobacterium spp
IJVT: Internal jugular vein thrombosis
LMWH: Low-molecular-weight-heparine
MRI: Magnetic Resonance Imaging
NR: Normal range
VTE: Secondary venous thromboembolism

## References

[1] Allen BW, Anjum F, Bentley TP. Lemierre Syndrome. In: StatPearls [Internet]. Treasure Island (FL): StatPearls Publishing; 2022 [cited 2023 Mar 4]. Available from: http://www.ncbi.nlm.nih.gov/books/NBK499846/.

[2] Valerio L, Zane F, Sacco C, Granziera S, Nicoletti T, Russo M (2021). Patients with Lemierre syndrome have a high risk of new thromboembolic complications, clinical sequelae and death: an analysis of 712 cases. J Intern Med.

[3] Hagelskjaer Kristensen L, Prag J (2008). Lemierre’s syndrome and other disseminated Fusobacterium necrophorum infections in Denmark: a prospective epidemiological and clinical survey. Eur J Clin Microbiol Infect Dis Off Publ Eur Soc Clin Microbiol.

[4] Riordan T (2007). Human infection with Fusobacterium necrophorum (Necrobacillosis), with a focus on Lemierre’s syndrome. Clin Microbiol Rev.

[5] Ridgway JM, Parikh DA, Wright R, Holden P, Armstrong W, Camilon F (2010). Lemierre syndrome: a pediatric case series and review of literature. Am J Otolaryngol.

[6] Holm K, Bank S, Nielsen H, Kristensen LH, Prag J, Jensen A (2016). The role of Fusobacterium necrophorum in pharyngotonsillitis - A review. Anaerobe.

[7] Brazier JS, Hall V, Yusuf E, Duerden BI (2002). Fusobacterium necrophorum infections in England and Wales 1990–2000. J Med Microbiol.

[8] Kuppalli K, Livorsi D, Talati NJ, Osborn M (2012). Lemierre’s syndrome due to Fusobacterium necrophorum. Lancet Infect Dis.

[9] El Chebib H, McArthur K, Gorbonosov M, Domachowske JB (2020). Anaerobic necrotizing pneumonia: another potential life-threatening complication of Vaping?. Pediatrics.

[10] Repper DC, Arrieta AC, Cook JE, Renella P (2020). A case of Lemierre Syndrome in the era of COVID-19: all that glitters is not gold. Pediatr Infect Dis J.

[11] Sheth SP, Ilkanich P, Congeni B (2018). Complicated Fusobacterium Sinusitis: a Case Report. Pediatr Infect Dis J.

[12] Unić J, Kovačić M, Jakovljević G, Batoš AT, Grmoja T, Hojsak I (2018). Lemierre Syndrome in adolescent with active Ulcerative Colitis. Pediatr Gastroenterol Hepatol Nutr.

[13] Monroe EJ, Amlie-Lefond CM (2018). Cone beam computed tomography-guided transpterygoidal aspiration of a carotid space abscess in Lemierre’s syndrome. Radiol Case Rep.

[14] Kumral AVW, Petersen WC, Heitz C, Waggoner-Fountain LA, Belyea BC (2017). Lemierre’s syndrome as a trigger for secondary hemophagocytic lymphohistiocytosis. J Pediatr Hematol Oncol.

[15] Bonhoeffer J, Trachsel D, Hammer J, Nava E, Heininger U (2010). Lemierre syndrome and nosocomial transmission of Fusobacterium necrophorum from patient to physician. Klin Padiatr.

[16] Horwitz M, Chaumoître K, Grimaldi C, Retornaz K, Nicaise C, Thomachot L (2013). Spontaneous regression of multiple Rasmussen aneurysms in a child with Lemierre syndrome and pulmonary abscesses. Pediatr Infect Dis J.

[17] Hawes D, Linney MJ, Wilkinson R, Paul SP (2013). Lemierre’s syndrome: the importance of early detection. Br J Nurs Mark Allen Publ.

[18] Waterman JA, Balbi HJ, Vaysman D, Ayres RA, Caronia CG (2007). Lemierre syndrome: a case report. Pediatr Emerg Care.

[19] de Lima JE, Levin M (2003). Lemierre’s syndrome: post-anginal septicemia. Pediatr Radiol.

[20] Busko JM, Triner W (2004). Lemierre syndrome in a child with recent pharyngitis. CJEM.

[21] Hoehn S, Dominguez TE (2002). Lemierre’s syndrome: an unusual cause of sepsis and abdominal pain. Crit Care Med.

[22] Klinge L, Vester U, Schaper J, Hoyer PF (2002). Severe Fusobacteria infections (Lemierre syndrome) in two boys. Eur J Pediatr.

[23] Brook I (2015). Fusobacterial head and neck infections in children. Int J Pediatr Otorhinolaryngol.

[24] Goldhagen J, Alford BA, Prewitt LH, Thompson L, Hostetter MK (1988). Suppurative thrombophlebitis of the internal jugular vein: report of three cases and review of the pediatric literature. Pediatr Infect Dis J.

[25] Baquero F, Reig M (1992). Resistance of anaerobic bacteria to antimicrobial agents in Spain. Eur J Clin Microbiol Infect Dis Off Publ Eur Soc Clin Microbiol.

[26] Armstrong AW, Spooner K, Sanders JW (2000). Lemierre’s syndrome. Curr Infect Dis Rep.

[27] Golpe R, Marín B, Alonso M (1999). Lemierre’s syndrome (necrobacillosis). Postgrad Med J.

[28] Chirinos JA, Lichtstein DM, Garcia J, Tamariz LJ (2002). The evolution of Lemierre syndrome: report of 2 cases and review of the literature. Med (Baltim).

[29] De Smet K, Claus PE, Alliet G, Simpelaere A, Desmet G (2019). Lemierre’s syndrome: a case study with a short review of literature. Acta Clin Belg.

[30] Sinave CP, Hardy GJ, Fardy PW (1989). The Lemierre syndrome: suppurative thrombophlebitis of the internal jugular vein secondary to oropharyngeal infection. Med (Baltim).

[31] Spaziante M, Giuliano S, Ceccarelli G, Alessandri F, Borrazzo C, Russo A (2020). Gram-negative septic thrombosis in critically ill patients: a retrospective case-control study. Int J Infect Dis IJID Off Publ Int Soc Infect Dis.

[32] Celikel TH, Muthuswamy PP (1984). Septic pulmonary emboli secondary to internal jugular vein phlebitis (postanginal sepsis) caused by Eikenella corrodens. Am Rev Respir Dis.

[33] Anton E (2007). Lemierre syndrome caused by Streptococcus pyogenes in an elderly man. Lancet Infect Dis.

[34] Camacho-Cruz J, Preciado H, Beltrán N, Fierro L, Carrillo J (2019). Lemierre’s syndrome caused by Streptococcus anginosus presenting as Postseptal Cellulitis in a Pediatric patient. ORL J Oto-Rhino-Laryngol Its Relat Spec.

[35] Stauffer C, Josiah AF, Fortes M, Menaker J, Cole JW (2013). Lemierre syndrome secondary to community-acquired methicillin-resistant Staphylococcus aureus infection associated with cavernous sinus thromboses. J Emerg Med.

[36] Osowicki J, Kapur S, Phuong LK, Dobson S (2017). The long shadow of lemierre’s syndrome. J Infect.

[37] Venglarcik J (2003). Lemierre’s syndrome. Pediatr Infect Dis J.

[38] Dagan R, Powell KR (1987). Postanginal sepsis following infectious mononucleosis. Arch Intern Med.

[39] Brook I (2005). The association of anaerobic bacteria with infectious mononucleosis. Anaerobe.

[40] Sacco C, Zane F, Granziera S, Holm K, Creemers-Schild D, Hotz MA (2019). Lemierre Syndrome: clinical update and protocol for a systematic review and individual Patient Data Meta-analysis. Hamostaseologie.

[41] Bansal AG, Oudsema R, Masseaux JA, Rosenberg HK (2018). US of Pediatric superficial masses of the Head and Neck. Radiogr Rev Publ Radiol Soc N Am Inc.

[42] Koo J, Pong A, Dory C, Farnaes L, Thornburg CD (2020). Management and outcomes of pediatric septic thrombophlebitis: a case series. Pediatr Hematol Oncol.

[43] Leugers CM, Clover R (1995). Lemierre syndrome: postanginal sepsis. J Am Board Fam Pract.

[44] de Benedictis FM, Kerem E, Chang AB, Colin AA, Zar HJ, Bush A (2020). Complicated pneumonia in children. Lancet Lond Engl.

[45] Yoshikawa S, Ueda T, Fujiwara T (2022). Use of intravascular hypo- and hyper-attenuation on non-contrast-enhanced computed tomography in diagnosing acute septic thrombophlebitis. J Radiol Case Rep.

[46] Goldenberg NA, Knapp-Clevenger R, Hays T, Manco-Johnson MJ (2005). Lemierre’s and Lemierre’s-like syndromes in children: survival and thromboembolic outcomes. Pediatrics.

[47] Patel PN, Levi JR, Cohen MB (2020). Lemierre’s syndrome in the pediatric population: Trends in disease presentation and management in literature. Int J Pediatr Otorhinolaryngol.

[48] Johannesen KM, Bodtger U (2016). Lemierre’s syndrome: current perspectives on diagnosis and management. Infect Drug Resist.

[49] Gore MR (2020). Lemierre Syndrome: a Meta-analysis. Int Arch Otorhinolaryngol.

[50] Kuhajda I, Zarogoulidis K, Tsirgogianni K, Tsavlis D, Kioumis I, Kosmidis C (2015). Lung abscess-etiology, diagnostic and treatment options. Ann Transl Med.

[51] Lee WS, Jean SS, Chen FL, Hsieh SM, Hsueh PR (2020). Lemierre’s syndrome: a forgotten and re-emerging infection. J Microbiol Immunol Infect Wei Mian Yu Gan Ran Za Zhi.

[52] Monagle P, Chan AKC, Goldenberg NA, Ichord RN, Journeycake JM, Nowak-Göttl U (2012). Antithrombotic therapy in neonates and children: antithrombotic therapy and Prevention of thrombosis, 9th ed: american college of chest Physicians evidence-based clinical practice guidelines. Chest.

[53] Goldenberg NA, Kittelson JM, Abshire TC, Bonaca M, Casella JF, Dale RA (2022). Effect of anticoagulant therapy for 6 weeks vs 3 months on recurrence and bleeding events in patients younger than 21 years of Age with provoked venous thromboembolism: the Kids-DOTT Randomized Clinical Trial. JAMA.

[54] Bush A, Lintowska A, Mazur A, Hadjipanayis A, Grossman Z, Del Torso S (2021). E-Cigarettes as a growing threat for children and adolescents: position Statement from the European Academy of Paediatrics. Front Pediatr.

